# Efficacy and safety of Yinchenwuling Powder for nonalcoholic fatty liver disease: A protocol for systematic review and meta-analysis of randomized controlled trials

**DOI:** 10.1097/MD.0000000000032088

**Published:** 2022-12-02

**Authors:** Peishan Wu, Jiaqi Zhang, Jingjing Xiao, Guangwen Huang, Jiahui Li, Zheng Zhou

**Affiliations:** a Dongguan Hospital of Guangzhou University of Chinese Medicine, Dongguan, China; b The Second Clinical Medical College, Guangzhou University of Traditional Chinese Medicine, Guangzhou, China.

**Keywords:** meta-analysis, nonalcoholic fatty liver disease, protocol, randomized controlled trial, systematic review, Yinchenwuling Powder

## Abstract

Nonalcoholic fatty liver disease (NAFLD) is the most common liver disease worldwide. Owing to the complexity of NAFLD pathogenesis, there is still no specific medication for NAFLD that is safe and effective. Yinchenwuling Powder (YCWLP), a classic Chinese formula, has been widely applied to NAFLD and its efficacy has been proven in numerous studies. However, systematic evaluation of the efficacy and safety of YCWLP for NAFLD is still lacking.

**Methods:**

We will search 8 databases to collect randomized controlled trials of patients with NAFLD treated in the YCWLP from the database inception to September 30, 2022. Two researchers will independently perform the selection of studies, data extraction, and assessment of the risk of bias. The Cochrane Review Manager (RevMan5.4) software will be used for data synthesis and analysis.

**Results:**

Comprehensive evidence of YCWLP for the treatment of NAFLD will be provided in this study.

**Conclusion:**

The efficacy and safety of YCWLP in treating NAFLD will be proved, providing feasible and effective clinical recommendations for the treatment of NAFLD.

## 1. Introduction

Nonalcoholic fatty liver disease (NAFLD) is a clinicopathological syndrome characterized by diffuse hepatocellular bullae steatosis and adipose tissue accumulation caused by nonalcoholic and other definite liver damage factors. The disease spectrum includes nonalcoholic simple fatty liver, nonalcoholic steatohepatitis, and nonalcoholic fatty cirrhosis. NAFLD has become the most common liver disease in the world. According to statistics, the overall global prevalence of NAFLD as of 2019 is approximately 25.2%.^[1]^ The prevalence of NAFLD among obese children is 26%,^[2]^ which indicates that the age of onset of NAFLD tends to be younger. However, the pathogenesis of NAFLD is still unclear and may be related to multiple pathways, such as insulin resistance, activation of lipid-stimulating factors, chronic inflammation, oxidative stress, mitochondrial damage, intestinal flora disorder, and genetic factors.^[3,4]^ Owing to the complexity NAFLD pathogenesis, there are still no targeted drugs for therapy. The recommended intervention is mainly lifestyle changes, such as dietary modification and appropriate exercise.^[5]^ Western medicine adjuvant therapeutic drugs include hepatoprotective drugs, insulin sensitizers, lipid-lowering drugs, antioxidants, and new synthetic drugs such as Obeticholic acid and Elafibrano, but they are still in the clinical trial stage, and their clinical efficacy has yet to be verified.^[6,7]^

Traditional Chinese medicine has potential advantages and development prospects for the treatment of NAFLD. Yinchenwuling Powder (YCWLP), which is composed of Artemisia capillaris Thunb, Alisma plantago-aquatica subsp. orientale, Poria cocos, Polyporus umbellatus, Atractylodes macrocephala Koidz, and Cinnamomum cassia, is frequently used for the treatment of NAFLD. YCWLP, derived from the Synopsis of Prescriptions of the Golden Chamber by Zhang Zhongjing, can invigorate the spleen, induce diuresis, and dispel dampness. In recent years, an increasing number of clinical reports and experiments have demonstrated the effectiveness of YCWLP in treating NAFLD. However, systematic evaluation of the efficacy and safety of YCWLP for NAFLD is still lacking. Therefore, following the principles of evidence-based medicine, this study will aim to conduct a systematic review and meta-analysis of the efficacy and safety of the YCWLP in the treatment of NAFLD to provide evidence-based medical evidence and clinical decision guidance for conducting relevant clinical studies.

## 2. Methods

### 2.1. Protocol and registration

This study has been registered with the International Register of Expectations System Evaluation PROSPERO (registration number: CRD42022345593). We will structure the protocol following the preferred reporting items for systematic reviews and meta-analyses. The publication of the final report will record any protocol changes during the implementation. The Preferred Reporting Items for Systematic Reviews and Meta-Analysis extension statement will be used to ensure all aspects of the reporting methods and results.

### 2.2. Eligibility criteria

#### 2.2.1. Participant

Referring to the diagnostic criteria of NAFLD consensus and guidelines,^[8–10]^ patients with a definite diagnosis of NAFLD were included as the research subjects, regardless of case source, sex, age, or disease duration, except for secondary fatty liver caused by alcoholic fatty liver, viral hepatitis, and autoimmune liver disease.

#### 2.2.2. Interventions/comparators

Interventions will include YCWLP without limits on the dosage form, frequency, and dosage. The intervention was only the YCWLP or YCWLP plus control group treatment. Comparators included blanks, placebo, Western medicine, lifestyle interventions, and exercise.

#### 2.2.3. Outcome measures

Main outcomes included the total effective rate, aspartate aminotransferase, alanine aminotransferase, triacylglycerol, and total cholesterol. Additional outcomes included the traditional Chinese medicine syndrome score scale, body mass index, fasting blood glucose level, ultrasound imaging changes, and the incidence of adverse events.

#### 2.2.4. Study design

Randomized controlled trials (RCTs) of YCWLP in the treatment of NAFLD will be comprehensively searched, regardless of the language or publication date. The literature included should be original articles of peer review, excluding reviews, case reports, animal trials, mechanistic studies, and other unpublished studies.

### 2.3. Search strategy

We will search the databases encompassing Cochrane Central Register of Controlled Trials, PubMed, Web of Science, Embase, Chinese National Knowledge Infrastructure, WanFang Data, Chinese Scientific Journals Database, and Chinese Biomedical Literature Database to collect RCTs of patients with NAFLD treated in YCWLP. The search time frame is from the database inception through September 30, 2022, and the search language is unlimited. The searches include journals, newspapers, conferences, and theses. The search method uses a combination of subject terms and free words, and we will adjust to the characteristics of each database when necessary. References incorporated into the study are also searched to supplement the access to relevant information. The search terms include Yin Chen Wu Ling Powder, nonalcoholic fatty liver, nonalcoholic steatohepatitis, and RCT. The search strategy is presented as follows taking the Web of Science as an example (Table 1).

**Table 1:** Search strategy of the PubMed.

Number	Search terms
#1	Nonalcoholic Fatty Liver Disease [MeSH Terms]
#2	Nonalcoholic Fatty Liver Disease [Title/Abstract]
#3	NAFLD [Title/Abstract]
#4	Nonalcoholic Fatty Liver Disease [Title/Abstract]
#5	Fatty Liver, Nonalcoholic [Title/Abstract]
#6	Fatty Livers, Nonalcoholic [Title/Abstract]
#7	Liver, Nonalcoholic Fatty [Title/Abstract]
#8	Nonalcoholic Fatty Liver [Title/Abstract]
#9	Nonalcoholic Fatty Livers [Title/Abstract]
#10	Nonalcoholic Steatohepatitis [Title/Abstract]
#11	Steatohepatitis, Nonalcoholic [Title/Abstract]
#12	#1 OR #2 OR #3 OR #4 OR #5 OR #6 OR #7 OR #8 OR #9 OR #10 OR #11
#13	Yinchenwuling [Supplementary Concept]
#14	YCL powder [Title/Abstract]
#15	#13 OR #14
#16	Randomized Controlled Trial [Publication Type]
#17	Controlled Clinical Trial [Publication Type]
#18	#16 OR #17
#19	#12 AND #15 AND #18

MeSH = medical subject headings.

### 2.4. Study selection and data extraction

**Figure 1.**
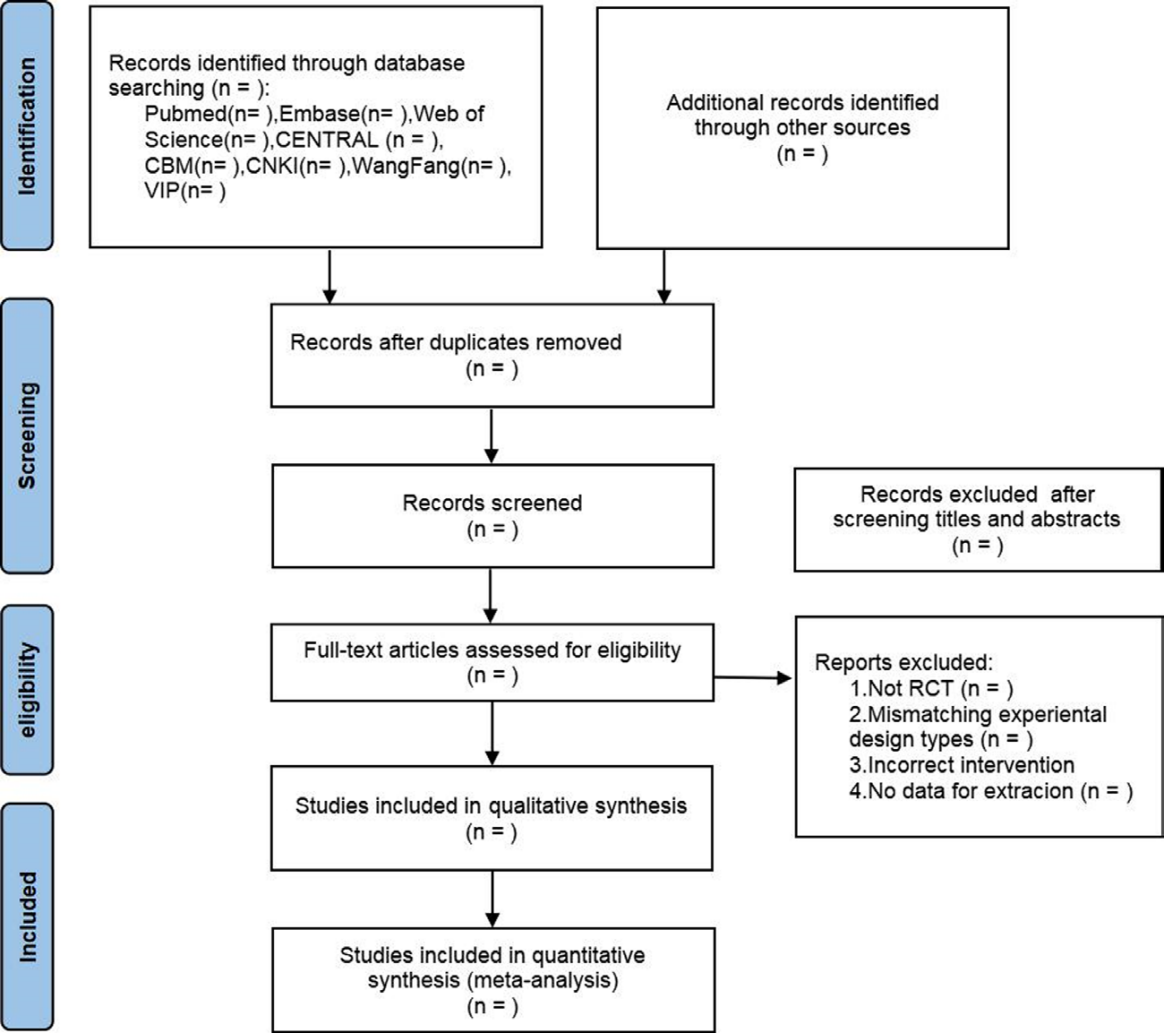
The Preferred Reporting Items for Systematic Reviews and Meta-Analysis (PRISMA) flow diagram to describe the search process.

Two researchers (W.P.S. and X.J.J.) will separately screen out the literature, extract information, and then cross-check it. In case of disagreement, we will discuss and negotiate. Otherwise, a third researcher (H.G.W.) will make the final decision. The literature will be screened by reading the title and abstract to exclude irrelevant literature at the beginning, and then downloading and reading the full text to determine whether to include it strictly according to the inclusion and exclusion criteria. A flow diagram of the search and selection processes is shown in Figure 1. Information will be extracted using a previously manufactured Excel sheet for the included literature, which includes the following content. Basic information about the included studies, such as the title, first author, and year of publication. Baseline characteristics of the participants, including the sample volume of each group, age, and disease duration. The specific intervention and control protocols, included drug name, dose, administration, and duration of treatment. Outcome measures concerned and adverse effects.

### 2.5. Quality assessment

Two researchers (W.P.S. and X.J.J.) will assess the methodological quality of the included studies according to the “bias risk assessment tool” in the Cochrane Handbook for Systematic Reviews.^[11]^ In case of disagreement, a third researcher (Z.Z.Q.) will assist in the adjudication. The main items of the assessment are as follows: random sequence generation, allocation concealment, blinding of participants and personnel, blinding of outcome assessment, incomplete outcome data, selective outcome reporting, and other potential biases. The results of each item will be divided into high-, low-, or unclear risk according to the specific situation of the included studies.

### 2.6. Statistical analysis

Statistical analysis will be conducted using RevMan 5.4 software provided by the Cochrane Collaboration. For dichotomous variables, we will use the odds ratio for analysis, whereas we will use the mean difference for continuous variables as effect sizes. A 95% confidence interval is used as a statistical indicator. We will consider the statistical difference when the *P* value is below .05. The degree of heterogeneity is determined according to the *P* value and *I*^2^. If *I*^2^ ≤ 50%, a fixed-effects model will be used to calculate the combined statistic. In the case of *I*^2^>50%, the heterogeneity will be considered high; therefore, we will further explore the sources of heterogeneity by subgroup analysis of studies. After excluding obvious clinical and methodological heterogeneity, a random-effects model will be used if heterogeneity remains. Based on the effects of the sample size, missing data results, and methodological quality, we will perform sensitivity analyses to check the robustness of the pooled results. We will use descriptive analysis when the data cannot be combined for other reasons.

### 2.7. Assessment of publication bias

When outcome measures are included in >10 studies, funnel plots will be used to analyze publication bias. If the funnel plot is not completely symmetrical, it suggests a potential risk of publication bias.

### 2.8. Ethics and dissemination

It is a protocol for a systematic review and meta-analysis of studies. Ethical approval is not required because no patient or public is involved in this study. The results of this systematic review and meta-analysis will be written as a manuscript and submitted to journals for the dissemination of information.

## 3. Discussions

With the increasing prevalence of obesity and metabolic syndrome, NAFLD has replaced viral hepatitis as the leading chronic liver disease worldwide. NAFLD can not only further develops into hepatitis, cirrhosis, and hepatocellular carcinoma, but is also closely related to metabolic syndrome, type 2 diabetes, atherosclerotic cardiovascular disease, colorectal tumors, and other systemic diseases. As a commonly used formula for the treatment of NAFLD, YCWLP has hepatoprotective, choleretic, anti-inflammatory, hypolipidemic, and insulin-resistant properties. Network pharmacology found that quercetin, beta-sitosterol, and sitosterol may be the key components of YCWLP in treating NAFLD.^[12]^ YCWLP may interfere with inflammation and immunity, metabolic homeostasis, lipotoxicity, hepatocyte death, and other mechanisms by modulating potential signaling pathways, such as NAFLD, cytokine-cytokine receptor interaction, and insulin resistance, suggesting that YCWLP plays a role in the treatment of NALFD through a multi-component, multi-target, and multi-mechanism approach.^[13]^ Animal experiments have shown that YCWLP can regulate lipid metabolism and slow down the degree of steatosis by downregulating the expression levels of RBP4 and chemerin adipokines, lowering total cholesterol, TG, and LDL-C levels, thereby reducing tissue inflammation and cell degeneration, and protecting hepatocytes, which act as a treatment for NAFLD.^[14]^ Clinical studies have also shown that YCWLP can improve clinical symptoms, reduce body weight, lower transaminases, regulate blood lipids, and improve hepatic steatosis and insulin resistance in patients with NAFLD.^[15]^ However, there is still a lack of evidence-based medical evidence supporting YCWLP treatment in patients with NASH. Therefore, we aimed to evaluate the efficacy and safety of YCWLP in the treatment of NASH through this systematic review and meta-analysis. However, this study still has some limitations, which may be due to the small sample size and low quality of the included literature. Differences in patient duration, age, dose form, route of administration, and dosage used in different studies are also one reason for limitations. Publication bias caused by the difficulty of publishing studies with negative results also leads to limitations. Nevertheless, this study is expected to provide feasible and effective clinical recommendations for the treatment of NASH and more reliable evidence for the use of YCWLP in treating NAFLD.

## Author contributions

All authors participated in drafting the protocol for this systematic review and approved the final manuscript. Peishan Wu, Jiaqi Zhang and Jingjing Xiao conceived the study and wrote the original draft. Zheng Zhou, Jiahui Li and Guangwen Huang revised it. Peishan Wu and Jiaqi Zhang developed the search strategies. Peishan Wu and Jingjing Xiao will independently accomplish the study selection and data extraction and assess the risk of bias. Guangwen Huang will be the arbitrator when meeting disagreements. Peishan Wu, Jiaqi Zhang and Guangwen Huang will perform the data syntheses. Zheng Zhou, as the corresponding author, will be responsible for overseeing every process of the audit review to control the quality of the study.

**Conceptualization:** Peishan Wu, Jingjing Xiao, Zheng Zhou.

**Data curation:** Peishan Wu, Jingjing Xiao.

**Formal analysis:** Peishan Wu, Jiaqi Zhang, Guangwen Huang.

**Funding acquisition:** Zheng Zhou.

**Project administration:** Guangwen Huang, Jiahui Li, Zheng Zhou.

**Writing – original draft:** Peishan Wu, Jiaqi Zhang.

**Writing – review & editing:** Jiahui Li, Zheng Zhou.
